# Response to Letter to the Editor Regarding the CALORI Trial

**DOI:** 10.1016/j.jscai.2025.102629

**Published:** 2025-03-11

**Authors:** Brian K. Mitchell, Anna Tomdio, Muhammad S. Pir, Suraj K. Mishra, Pradeep Dayanand, Guillaume Bonnet, Maria C. Alu, Zachary M. Gertz

**Affiliations:** Division of Cardiovascular Medicine, Virginia Commonwealth University, Richmond, Virginia; Mary Washington Hospital, Fredericksburg, Virginia; Division of Cardiovascular Medicine, Virginia Commonwealth University, Richmond, Virginia; Division of Cardiovascular Medicine, Virginia Commonwealth University, Richmond, Virginia; Allegheny Health Network St. Vincent Hospital, Erie, Pennsylvania; Clinical Trials Center, Cardiovascular Research Foundation, New York, New York; CHU de Bordeaux, Bordeaux, France; Clinical Trials Center, Cardiovascular Research Foundation, New York, New York; Division of Cardiovascular Medicine, Virginia Commonwealth University, Richmond, Virginia

We appreciate the opportunity to respond to the critique of our study.^1^

The writers mention variability in oral intake protocols. We did not have any variability in our protocol, which allowed patients to eat ad lib until called to the cardiac catheterization laboratory. This is the same protocol used in several recent randomized trials, including the recently published TONIC trial.^2^^,^^3^ We believe this practice is best for patients and simplest to implement in routine practice.

The writers bring up our exclusion of high-risk groups. We excluded hemodynamically unstable patients as well as those requiring mechanical circulatory support. We also allowed for physician discretion to exclude patients considered to be at particularly high risk. This, again, is similar to what other recent trials have done.^3^ We did this because patients at the highest risk of destabilization may require urgent intubation, and fasting prior to intubation is still routine practice for elective cases.

Lastly, the writers mention our single-center design. While this is common (other trials on this topic have been single-center as well), we agree that datasets including multiple institutions may be necessary prior to changing practice on a larger scale. With that in mind, we applaud ongoing efforts to complete patient-level meta-analyses combining the multiple trials that have been completed and published in the past few years. We agree that combining the findings from several centers may provide enough evidence to end fasting as a routine practice prior to cardiac catheterization.
